# The Central Park

**Published:** 1863-08

**Authors:** 


### HALL'S JOURNAL OF HEALTH.

Our Legitimate Scope is almost boundless: for whatever begets pleasurable and harmless feelings, promotes Health; and whatever induces disagreeable sensations, engenders Disease.

WE AIM TO SHOW HOW DISEASE MAY BE AVOIDED, AND THAT IT IS BEST, WHEN SICKNESS COMES, TO TAKE NO MEDICINE WITHOUT CONSULTING A PHYSICIAN.

Vol. X.] AUGUST, 1863. [No. 8.

### THE CENTRAL PARK.

On the twenty-seventh day of June, 1863, at six and a half o'clock in the afternoon, we counted, within five minutes, watch in hand, from our study-window, overlooking Fifth Avenue, at a point just one half a mile from its main entrance, one hundred and ten pleasure-vehicles pass, not including gentlemen and ladies on horseback, making over thirteen hundred in a single hour, on the chief drive to "the Park," which is the name given "for short" by practical New-Yorkers, to the magnificent pleasure-grounds of the great Metropolis; destined to be the "lungs," and a great fountain of health for the teeming thousands of the chief city of the Empire State. And as every reader of these pages who has ever visited the Central Park would love to see it again; and those who have not driven through it, or promenaded its delightful walks, may one day do so, if they have even a trace of taste or admiration for what is splendid and beautiful, it will doubtless add to the gratification of the many, to be able to have at least a synopsis of the chief items of interest connected with this great enterprise, as taken from official sources, to wit, the report of 1862, furnished by the courtesy of the Hon. Thomas C. Fields, the Secretary of the Board of Commissioners, and one of its Auditing Committee.

It may aid the memory by dealing mainly in round numbers.

The Central Park contains 843 acres.

The land alone cost nearly $4500 an acre, or $3,788,751.

Improvements to January 1st, 1863, $3,583,674.

Total cost to January 1st, 1863, $7,372,425.

Our rural cousins will exclaim, "What an expensive whistle!" and may straightway conclude that "it cost more than it comes to," which means that it is more than it is worth. But that is a mistake. It does not cost the present generation of New-Yorkers one cent. The expense has been saddled on posterity, on the very plausible ground, that as we have the trouble of building it, our children ought to pay for it, especially as they only can have the enjoyment of it in its matured beauty and magnificence. In short, the money was borrowed to be paid in a decade or two hence; the present generation only taking care of the interest; but even that is met by the taxes derived from the increased worth of the lots adjoining the Park. The assessed value of the three wards which surround the Park, in 1856, before it was commenced, was $26,000,000. The increased value at this time is about $25,000,000 more; yielding the city in taxes sufficient, with certain rentals, to pay the interest on the Park debt.

The largest number of workmen employed on the Park at any one time was 3666.

The average daily force during one year was 3000.

As the workmen are employed and paid by the day, they can only work in good weather. The average number of working days in a month was 21.

Average number of working days during a year, 260.

Up to January 1st, 1862, the number of cubic yards of earth, etc., removed was 2,000,000; of cubic yards of rock excavated, 250,000.

The number of brick used to that time was 5,000,000.

The total number of plants, vines and deciduous trees set out was 53,000.

The highest point of the Park is at Eighty-third street, and is 140 feet above tide-water. The lowest point is a few inches below tide-water, at One Hundred and Seventh street.

The new reservoir covers one hundred and six acres; is about thirty feet deep, holds a thousand millions of gallons of water, and cost a million and a half of dollars. Around its rim is a walk for pedestrians; outside of that is a bridle-path, and beyond that again is a beautiful carriage-drive.

Five miles of bridle-path have been completed; eight miles of carriage-road; eighteen miles of foot-path.

It is a five-mile drive from the Battery, or lower part of the city to the nearest Park-gate, which is in Fifty-ninth street. The Park is bounded by two parallel lines, east and west, of Fifth and Eighth avenues, and is half a mile broad by nearly three miles long.

The Central Park was originally laid out to extend north and south between Fifty ninth and One Hundred and Sixth streets, but it was afterward found that for completeness, it should reach to One Hundred and Tenth street, four blocks farther, or one fifth of a mile. And now for a digression, for the purpose of contemplating a phase of human nature. This desired addition was mainly a hill of rock and a swamp; hence was of comparatively little value; still it was private property, and the Legislature empowered the Supreme Court to appoint several gentlemen who should be "good and true," to say what the land was worth. Such men were selected as were considered above reproach and unpurchasable. Eighteen weary months were spent in coming to a conclusion; some men would have finished it up in as many days, as a whole community were interested in its prompt settlement. The decision arrived at was that this rock and piece of swamp, which had been running to seed since the days of Adam, was worth a million and a half of dollars, about half as much as the entire Park besides, which was two miles nearer the city, and contained over ten times as much land. The report was such an absurdity, that the contemplated plan of addition was abandoned. It did not appear that any of these commissioners owned any of the land, nor that they had any relatives or friends or partners at all interested in the matter. It may have been an error in judgment. But they had been to some cost of time, trouble, and money in ascertaining what the swamp and rock were worth, and it was very proper that they should be paid for the same; when asked to name the amount for their services, they put it down at the sum of $73,335.52. Over half of this amount was claimed by nine men for personal services of themselves, except the surveyor, who must have had assistants. The people and the press made such a racket about this bill of costs, that the Supreme Court decided that it could not decide as to its propriety, but selected an unpurchasable man as referee, the Hon. John B. Haskin, who reported to the court that the charges were "extravagant and unreasonable, and that this court should make an order vacating, annulling, and setting aside said bill of costs, and every part and parcel thereof." The commissioners then had leave to correct their bill, which they did, and struck off twenty thousand dollars; but the court and referee thought it was still too much by twenty-five thousand, and fixed it at the sum of thirty thousand dollars; less than half the original sum. In connection with this transaction, it is suggestive to notice the conduct of certain other parties.

The widow of the lamented Crawford, the sculptor, has generously bestowed eighty-seven casts in plaster for the ornamentation of the Park. R. K. Haight, Esq., has, with great liberality, donated the statue of Flora, the *chef d'œuvre* of its eminent author. Our fellow-citizen, the Hon. Auguste Belmont, has also made a liberal contribution in the same direction.

### ANIMALS IN THE PARK.

The nucleus of a collection for the study of natural history "from life," is already found in contributions from the liberal-minded at home and abroad.

1860, May 24th.—Twelve white swans, presented by the Senate of the city of Hamburgh.

October 18th.—Twenty-four white swans, presented by the worshipful company of Vintners, London.

Twenty-six white swans, presented by the worshipful company of Dyers, London.

November 1st.—Ten white swans, presented by the city of Hamburgh.

Two trumpet cranes, presented by G. Granville White, Esq. One peacock, do.

One American eagle, presented by Albert S. Joslyn.

One deer, presented by Joseph Conrad.

One deer.

Gold fish, presented by Wm. D. Murphy, Esq.

Two Canadian geese, presented by Charles A. Graham, Esq.

### PARK CONSERVATORY.

Responsible parties have secured the right to construct and keep in order an immense conservatory, from which visitors may obtain, at a reasonable charge, the flowers and fruits of all countries, climes, and seasons, fresh from the bush and tree.

### MUSIC IN THE PARK.

Every Saturday afternoon, from May to November, when the weather is suitable, a large company of accomplished instrumental performers are engaged by the liberality of the Third and Sixth Avenue Railroad Companies, to discourse the melody of sweet sounds to delighted listeners; more than three thousand of whom were estimated to have been present on the last Saturday of May, which was the "opening day" of the present season, described by one of the daily papers thus:

"Agreeably to the announcement which appeared in our yesterday's issue, the music season commenced yesterday afternoon in the Park. The midday storm had somewhat prevented the usual large assemblage from being present; nevertheless a goodly number of persons arrived around the newly painted and gilded music-stand, which in itself, for its beauty alone, is an object of attraction. Ladies on horseback, in carriages, and on foot, were to be seen enjoying the music, and numbers of wounded soldiers reclined on the grass around the temple of Apollo, the natural carpet having been thrown open for public use. The birds could both be seen and heard during the intervals between the pieces, and the flowers and sheep, with the absence of all things usually seen in the busy, money-making world, would almost tempt the visitor to believe that he was actually in the country, instead of the center of Gotham. The rain had freshened up the vegetation and made it look charming. Every tree, plant and shrub is now in full bloom, either of leaf or flower, and the rhododendrons in the Ramble are exceedingly beautiful. A row on the lake exhibits new charms and attractions, among others that of the swans and other water-fowl engaged in the duty of incubation. The pleasure-boats are well conducted, comfortable, and hired out at reasonable rates. The Casino is now in progress and will soon be erected.

The Park authorities announce it as their intention to throw open the lawn to Sunday-school parties and picnics on application to the commissioners. On Friday last the Free Academy had the use of the ball-ground. It is the desire of the Managers of the Park that schools and children should enjoy the use of the grass when it is not detrimental to the beauty and progress of the Park.

### SKATING IN THE PARK.

This is the most popular, the most largely, and most joyously patronized of all the amusements of the Park, and the facilities of enjoyment afforded, have caused it to be considered both a healthful and graceful accomplishment all over the country. Seven years ago, the sight of a pair of skates on sale in a shop-window was a rarity. They may now be seen on a winter's day by thousands. The number of skating-days in a year varies from twenty to forty; and it has been estimated that as many as ten thousand persons have been seen on the ice at a time at "the Lake," which covers twenty acres, and presents one of the most delightfully animated panoramas which can be conceived of.

To show the thoughtfulness of the Managers of the Park, and as an evidence of their own vivid remembrance of the pastimes of childhood, arrangements have been made for the boys to enjoy the sport of "coasting," or riding down-hill in little hand "sleds."

The floor-like surfaces of the Park drives afford the most delightful sleighing for a great part of the winter, after the snow has once fairly covered the ground; then it is continuous until the final thaw of the early spring, as the snow is not cut up, and fouled by wagons, carts, and other business vehicles; for these are not allowed to enter the Park on any pretense, except through under-ground and out-of-sight roads; so that the Park has its attraction for winter as well as summer, and is destined to afford an incalculable amount of pleasure to those now living; but greater still to generations to follow, when it will be enjoyed in its more mature beauty.

Not one of the least gratifications connected with a visit to the Park is the police regulations; dressed in a gray uniform, the police are stationed in every portion of the Park, to prevent depredations on the shrubbery and instantly correct any disorder on the part of thoughtless or momentarily excited persons; to check fast driving and riding, so that nothing may occur to mar the pleasure or safety of an excursion to the Park. Great care is taken to exclude wagons, or persons carrying bundles, or drunken people. And in case of accident to vehicle or horse, the police are always at hand to give all the assistance possible or required; and ready also, to give courteously, any desired information.

The community were fortunate in the appointment of the gentlemen who should have the control over the construction of the Park, and over the expenditure of the millions which it would require to complete the great work. Well have they performed their high trusts, and their names are worthy of being placed on public record; they are, Charles H. Russell,

Thomas C. Fields, R. M. Blatchford, J. F. Butterworth, A. H. Green, Waldo Hutchins, H. G. Stebbins, Moses H. Grinnell, and Mr. E. P. Barker, Assistant Secretary, to whose ready courtesy we are indebted for statistical items in advance of the sixth annual report, for the year ending with December 31st, eighteen hundred and sixty two.

### BOATING IN THE PARK.

Omnibus-boats give a ride around the lake of twenty acres for ten cents. "Call-boats" for private parties, admitting but six persons, are had at reasonable rates.

### ACCESS TO THE PARK.

The Sixth Avenue cars leave the Astor House every two minutes, and for five cents convey you to the entrance of the Park on Fifty-ninth street, where carriages, holding four or more persons, will spend an hour in riding around and about the Park for two dollars. Vehicles for one or two are less. The Third Avenue cars leave the front of the Astor House every two minutes, and for six cents convey passengers to Seventy-first street, whence they can walk across to the Park, half a mile distant. Those who take this route can more readily walk over the Park, being put down near a central position; but we have not seen accommodation-carriages at that point.

It is interesting to notice the varying character of the persons who visit the Park at different times. The comers before breakfast are chiefly ladies and gentlemen on horseback; we see them from our study-window as early as four and a half o'clock of a summer morning. Those who come after breakfast may be classed generally as strangers in the city; these come in vehicles. About four o'clock of a May afternoon, the head of the cavalcade of fashion is first seen increasing in number steadily and rapidly, for about two hours; there are a few on horse, but the great body are those whose liveried coachmen and splendid span, and faultless turn-outs, plainly show them to be the *élite*, the millionaires, the aristocrats of the hour.

But the "great exhibition," we are sorry to say, is on Sundays. Thither thousands, and tens of thousands (sometimes) of pedestrians wend their way. The most numerous class is the quiet, economical German, alone, or with his wife and children; respectable and worthy citizens, accustomed to spend at least a portion of the Sabbath in this way, in the fatherland, they think it no harm. Americans who walk out to the Park on Sundays are mostly, to all appearance, of a low class, wild, rough, and rowdyish. Now and then a respectable-looking man is met going or returning, but he is either an invalid, a stranger, or some person of no special social position, usually.

The riders to the Central Park on Sundays embrace all classes of society, except the better class of citizens. In a mile and a half walk on Fifth Avenue, from dwelling to church, on a Sabbath afternoon, requiring less than half an hour, we have repeatedly counted over two hundred vehicles. But we do not remember to have seen a liveried turn-out, or to have recognized a respectable New-Yorker. The vehicles are of every size, age, shape, and pattern under the sun, holding from one person to a dozen; but whether sulky, barouche, gig, phaeton, or carriage, all have the "hack" look, showing they were hired, consequently were occupied by strangers, clerks, apprentices, journeymen, and fast young men generally; further indicated by the inevitable segar stuck in the mouth, slouched hat, boisterous mirth, a commonish look in the face, and a rowdy demeanor generally. Such, at least, is the impression left on the mind after they have passed along. The Sunday visitors to the Park, whether on foot, horse, or carriage, are evidently not the most elevated and refined members of our community.

The position of the most interesting and prominent localities of the Park is as follows:

Fifty-ninth street is the southern or nearest the city proper boundary of the Park.

Sixty-third street passes centrally an open ground of ten acres.

Sixty-eighth street crosses the beautiful "green" of fifteen acres, also the splendid "Mall."

Seventy-fourth street divides the skating and rowing lake of twenty acres; also the site of ornamental water, in connection with the Conservatory.

Seventy-seventh street crosses centrally the lovely "Ramble" of thirty-six acres.

Eighty-third street divides the old reservoir of thirty-five acres.

Ninety-first street bisects east and west the new reservoir.

One Hundredth street crosses an open ground of twenty-three acres.

The Central Park was laid out by Frederick Law Olmstead, a native of Hartford, Connecticut, after having visited the chief pleasure-grounds of Europe, and we may well suppose that it combines the beauties of them all. Lieutenant E. L. Viele, now General Viele, of the Union Army, was the original Engineer-in-Chief, which office is now ably filled by William H. Grant, Esq.

By all means let that portion of the Park be visited as a matter of suggestive interest, lying between One Hundred and Sixth and One Hundred and Tenth streets, embracing an area of seventy-three acres, containing a rock and a swamp; for surveying which, somebody charged the city thirty-two thousand dollars.

Now, in the heat of summer, the more knowing ones arrange to reach the Park about sun-down, and even later, leaving it about nine o'clock, thus avoiding the crowd and dust and heat of the afternoon, and breathing the cool, refreshing breeze which comes from the broad Atlantic, but a few miles distant, without having to pass over the dense and business part of the city; it is literally the breeze from the sea, all pure and fresh, which visits the Central Park on these beautiful summer evenings. Being near by, we have visited it after night, and find it the resort of families on foot, of gentlemen and ladies, who pass hours together in the most delightful promenades along the sandy shore of some quiet lake, or in some secluded by-path, under low hanging trees; next emerging all at once into a rustic summer-house, fanned by the evening winds; the lights of the distant city shining like so many diamonds of the first water; the stars glitter in the blue sky above, the moonbeams dance on the bosom of the placid lake and clattering rivulet; the cool air is loaded with the scent of sweetest flowers and reverberates the diapasons of frogs; while whispers soft and low, but more luscious than molasses, tell that love is there.

